# Heterodyne sensing of microwaves with a quantum sensor

**DOI:** 10.1038/s41467-021-22714-y

**Published:** 2021-05-12

**Authors:** Jonas Meinel, Vadim Vorobyov, Boris Yavkin, Durga Dasari, Hitoshi Sumiya, Shinobu Onoda, Junichi Isoya, Jörg Wrachtrup

**Affiliations:** 1 3rd Institute of Physics, University of Stuttgart Institute for Quantum Science and Technology IQST, Stuttgart, Germany; 2 Max Planck Institute for Solid State Research, Stuttgart, Germany; 3 Advanced Materials Laboratory, Sumitomo Electric Industries Ltd., Itami, Japan; 4 Takasaki Advanced Radiation Research Institute, National Institutes for Quantum and Radiological Science and Technology, Takasaki, Japan; 5 Faculty of Pure and Applied Sciences, University of Tsukuba, Tsukuba, Japan

**Keywords:** Electrical and electronic engineering, Quantum metrology

## Abstract

Diamond quantum sensors are sensitive to weak microwave magnetic fields resonant to the spin transitions. However, the spectral resolution in such protocols is ultimately limited by the sensor lifetime. Here, we demonstrate a heterodyne detection method for microwaves (MW) leading to a lifetime independent spectral resolution in the GHz range. We reference the MW signal to a local oscillator by generating the initial superposition state from a coherent source. Experimentally, we achieve a spectral resolution below 1 Hz for a 4 GHz signal far below the sensor lifetime limit of kilohertz. Furthermore, we show control over the interaction of the MW-field with the two-level system by applying dressing fields, pulsed Mollow absorption and Floquet dynamics under strong longitudinal radio frequency drive. While pulsed Mollow absorption leads to improved sensitivity, the Floquet dynamics allow robust control, independent from the system’s resonance frequency. Our work is important for future studies in sensing weak microwave signals in a wide frequency range with high spectral resolution.

## Abstract (web summary)

High-resolution microwave detection with NV centers in diamond is currently applicable to signals with frequencies below 10 MHz, thus limiting their range of applications. Here, the authors demonstrate detection of GHz signals with sub-Hz spectral resolution, not limited by the quantum sensor lifetime.

## Introduction

Precise detection of microwave frequency fields is of importance for a wide range of applications in cosmology^1^, radar^2,3^, quantum optics with quantum circuit systems^4–6^ and electron spin signals or coupling to phonons^7^. Oscillating magnetic fields, described by their amplitude and frequency, require a sensor with high sensitivity and high spectral resolution over a wide range of frequencies.

**Fig. 1:**
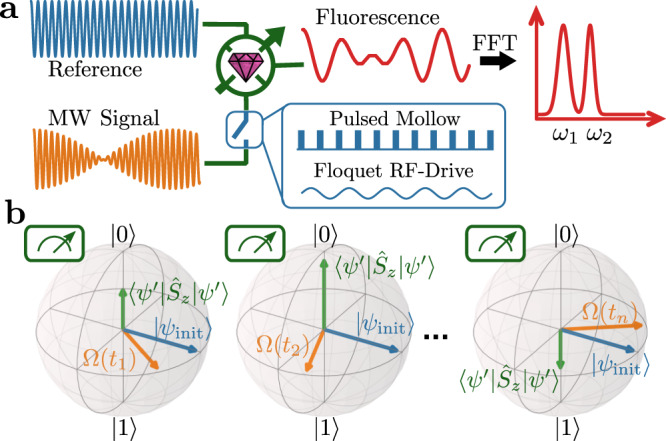
Microwave heterodyne detection principle and its realization with a quantum sensor. **a** Heterodyne detection of a multimode microwave frequency field relative to an external frequency reference using the NV center sensor as a mixer. The interaction with the signal is controlled by application of dressing fields. We benchmark two techniques: (1) pulsed Mollow absorption using dynamical decoupling sequence and (2) Floquet dynamics under strong RF-driving. The demodulated signal leads to high spectral resolution. **b** Heterodyne detection by creating the same initial state $\left|{\psi }_{{\rm{init}}}\right\rangle$ (blue) from a coherent external MW-source between sequential measurements. A long coherent signal Ω(*t*) (orange) is stroboscopically observed in the rotating frames. Finally, the z-projection (green) of $\left|{\psi }^{\prime}\right\rangle$, the final state after evolution under Ω, is measured, allowing to reconstruct the relative microwave phase.

Atomic systems, such as nitrogen vacancy (NV) centers in diamond, offer a microwave (MW) sensing platform through the electron spin transition^8–10^. Furthermore, the spin state can be optically pumped, leading to an effective sensor temperature of about 10 mK (99% polarization)^8^, hence in principle being able to resolve single photon level signals^11^. However, for any quantum sensors, the spectral resolution and sensitivity are linked through the lifetime of the system^12–14^. Advancements in experimental control of dynamical decoupling sequences allowed to separate sensitivity from spectral resolution^15,16^, widely applied in nuclear magnetic resonance (NMR) detection using NV-centers, e.g.,^17,18^. While with these techniques, radio frequency signals can be detected with a spectral resolution below 1 Hz, they fail for frequencies beyond 10 MHz. Thus, MW detection protocols based on the absorption in the electron spin transition^19^, though they could potentially reach high sensitivity^8,10^ and large spectral range^9^, are limited in spectral resolution with ${T}_{2}^{* }$ and *T*_1*ρ*_ times of the electron spin. In our work, we aim to overcome the resolution limitations and benefit from state-of-art sensing protocols for MW detection. Conceptually, we extend heterodyne detection^15,16^ to the microwave regime. Furthermore, we apply the sensitivity advancements brought by dynamical decoupling^8–10^ for MW sensing to heterodyne MW sensing. Finally, we profit from the robustness of Floquet dynamics for MW sensing^20–22^ and implement them as control fields for our protocols. This leads to a heterodyne MW sensor scheme, shown in Fig. 1a. We mix an external reference with the MW signal and detect a demodulated signal in the fluorescence of the NV-center.

This is achieved by creating the initial state of the sensor, using the above-mentioned coherent external reference MW source. This state evolves under the signal field, and is sensitive to the relative phase between reference and signal. We further show that the interaction with the signal field can be controlled with dressing fields while preserving the phase sensitivity. First, we study pulsed Mollow absorption, a dynamical decoupling sequence applied to sense MW-fields, making our protocol compliant with high sensitivity detection schemes and still achieving sensor-unlimited spectral resolution. Second, we study Floquet dressed states under strong longitudinal radio frequency (RF) drive, allowing to create detection sidebands independent from the resonance frequency of the quantum system and allowing to control the interaction strength.

## Results

### Theory

In the following, we theoretically describe how one achieves heterodyne sensing using a quantum sensor, here with NV center electron spin. The task at hand is to sense an oscillating microwave field with frequency *ω*, given by:

1 $${{\Omega }}(t,{\phi }_{0})={{{\Omega }}}_{0}\cos (\omega t+{\phi }_{0}).$$

Where Ω_0_ = *γB*_signal_ is the amplitude of the field with *γ* being the electron gyromagnetic ratio, and *ϕ*_0_ is the initial phase of the signal. The coupling of such a field to the electron spin of the NV center, is given by the Hamiltonian $H={{\Omega }}(t)\ \left({\boldsymbol{\theta }}\cdot \hat{{\bf{S}}}\right)$, where $\hat{{\bf{S}}}$ is the vector of spin operators for the triplet ground-state spin configuration of the NV center and ***θ*** is the direction of the microwave field relative to the NV axis (see Supplementary Fig. 3). The NV energy eigenstates with aligned magnetic field are defined as $\left|1\right\rangle ,\left|0\right\rangle$ and $\left|-1\right\rangle$. The contribution of longitudinal components of Ω(*t*, *ϕ*_0_) can be neglected for small amplitudes (*ω* ≫ Ω_0_), hence we assume Ω(*t*, *ϕ*_0_) to be along the x-axis. In secular approximation, the overall Hamiltonian becomes:

2 $$H={\omega }_{s}\hat{{S}_{z}}+{{{\Omega }}}_{0}\cos (\omega t+{\phi }_{0})\hat{{S}_{x}},$$

where *ω*_*s*_ is the transition frequency in the two-level subspace of the NV-spin triplet, ${\hat{S}}_{z}=({\sigma }_{z}-{\mathbb{1}})/2$ and ${\hat{S}}_{x}={\sigma }_{x}/\sqrt{2}$, where *σ* are the Pauli matrices and $\mathbb{1}$ is the identity. After transformation into the rotating frame, we get:

3 $$H^{\prime} ({\phi }_{0})=\frac{{{\Delta }}\omega }{2}\hat{{\sigma }_{z}}+\frac{{{{\Omega }}}_{0}}{2\sqrt{2}}\cos ({\phi }_{0})\hat{{\sigma }_{x}}+\frac{{{{\Omega }}}_{0}}{2\sqrt{2}}\sin ({\phi }_{0})\hat{{\sigma }_{y}},$$

where Δ*ω* = *ω*_*s*_ − *ω*, depicted as orange arrow in Fig. 1b. For heterodyne sensing, we have to add a second microwave field Ω_ref_(*t*, *ϕ*_ref_) acting as a reference. In our concept, the reference source recreates the initial state with a *π*/2 pulse:

4 $$\left|{\psi }_{{\rm{init}}}({\phi }_{{\rm{ref}}})\right\rangle =(\left|0\right\rangle +{e}^{i{\phi }_{{\rm{ref}}}}\left|\, - \,1\right\rangle )/\sqrt{2},$$

which evolves under the Hamiltonian in Eq. (3) for the time *τ*:

5 $$\left|\psi (\tau )\right\rangle =\hat{U}(H^{\prime} ({\phi }_{0}),\tau )\left|{\psi }_{{\rm{init}}}({\phi }_{{\rm{ref}}})\right\rangle .$$

After evolution, we measure the expectation value $\langle \hat{{S}_{z}}\rangle$:

6 $$\langle {S}_{z}(\tau ,{\phi }_{{\rm{ref}}},{\phi }_{0})\rangle \approx {{{\Omega }}}_{0}\tau \sin ({\phi }_{0}-{\phi }_{{\rm{ref}}})$$

for ${{\Omega }}^{\prime} \tau ,\frac{{{\Delta }}\omega }{{{\Omega }}^{\prime} }\ll 1$ with ${{\Omega }}^{\prime} =\sqrt{{{\Delta }}{\omega }^{2}+{{{\Omega }}}^{2}}$. We thus achieve a heterodyne response as we compare the phases of two microwave fields through the measurement of the ${\hat{S}}_{z}$ expectation value. We now perform a series of measurements at times *t*_*n*+1_ = *t*_*n*_ + *T* (sampling interval *T*), where the initial phases for each measurement are *ϕ*_0,*n*+1_ = *ϕ*_0,*n*_ + *ωT* and *ϕ*_ref,*n*+1_ = *ϕ*_ref,*n*_ + *ω*_ref_*T*. Experimentally and theoretically, it is instructive to analyze the autocorrelation *C*(*n*) between the single measurement outcomes *S*_*n*_ = *S*_*z*_ (*t*_*n*_) given by:

7 $$C(n)	= \, \langle {S}_{n^{\prime} }{S}_{n^{\prime} +n}\rangle \\ 	 = \, \mathop{\sum }\limits_{n^{\prime} =1}^{M}{{{\Omega }}}^{2}{\tau }^{2}\sin (\delta \omega Tn^{\prime} +\delta \phi )\sin (\delta \omega T(n^{\prime} +n)+\delta \phi )\\ 	 \approx \,\frac{1}{2}M{{{\Omega }}}^{2}{\tau }^{2}\cos (\delta \omega Tn),$$

where *δω* = *ω*_ref_ − *ω* is the demodulated frequency, *M* is the number of measurements and *δϕ* = *ϕ*_ref_ − *ϕ*_0_ is the initial phase difference. The above expression gives the autocorrelation of the $\hat{{S}_{z}}$ operator; in experiments, we will obtain the autocorrelation of the fluorescence photon counts collected in each measurement during the spin readout of the NV center. They can be further linked through the fluorescence spin contrast and average fluorescence of the NV center^23^.

In the discussion above, we assumed a segmented evolution, starting with the state preparation by the reference followed by the free evolution under the signal field. In experiments, a MW field is constantly interacting with the sensor. To achieve tunable interaction, we further apply dressing fields. This disables the sensing field’s influence during sensor state preparation (Ω_0_/Δ*ω* ≪ 1) and switches it on during the interaction when the dressing field is applied ((Δ*ω* − Ω_dressing_)/Ω_0_ ≪ 1). Here, Ω_dressing_ is the energy shift due to the dressing field (see Supplementary Notes 2–4 respectively for the analytical derivation). This leads to a Hamiltonian as described in Eq. (3).

### Heterodyne detection of microwave fields

**Fig. 2:**
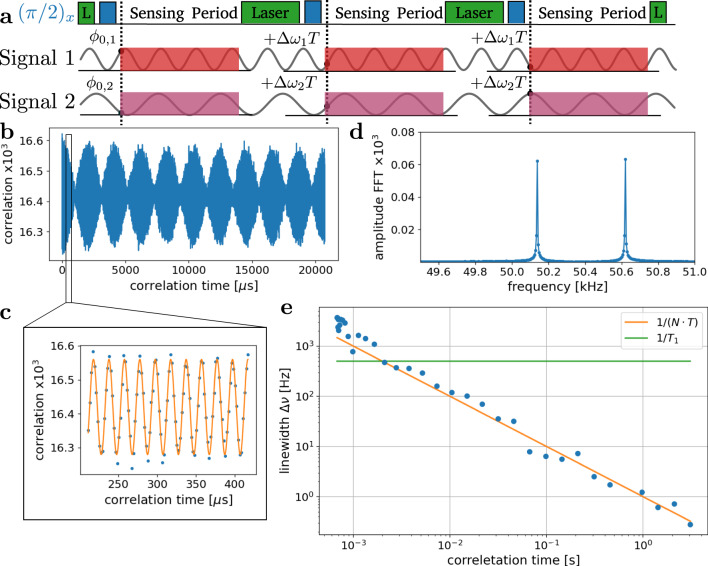
Heterodyne detection of microwave fields by phase-dependent evolution. **a** The NV center is initialized and readout with a green laser pulse (green), the external reference prepares the rotating frame by a *π*/2 pulse (blue) in which the signals (1*&* 2) are measured. The phase of the external source evolves relative to the signal 1 (Δ*ω*_1_ = 2*π* ⋅ 50.138 kHz) and signal 2 (Δ*ω*_2_ = Δ*ω*_1_ + 2*π* ⋅ 482 Hz) with Δ*ω*_1,2_Δ*t* between two measurements. In (**b**) autocorrelation of the measured photon counter time trace. The beating between the two signals can be clearly recognized, consisting of fast oscillations shown in (**c**). **d** Fourier spectrum of the autocorrelation. In (**e**) linewidth of the FFT peak as a function of the correlation length. The 1/(*N* ⋅ *T*) scaling of the linewidth shows the Fourier limited linewidth for the applied sinusoidal signal. For the longest correlation time of 3 seconds, a linewidth of 300 mHz is extracted, well below the sensor lifetime of 1/*T*_1_ ≈ 500 Hz.

We experimentally realize the heterodyne measurements by the scheme shown in Fig. 2a. The NV spin is initialized in the $\left|0\right\rangle$ state with a green laser pulse, then the superposition state $|{\psi}_{{\rm{init}}}({\phi}_{{\rm{ref}}})\rangle =\frac{1}{\sqrt{2}}(|0\rangle -{e}^{i{\phi }_{{\rm{ref}}}}|-1\rangle )$ is created by applying a $\left(\frac{\pi}{2}\right)$ pulse from a coherent MW-source. The phase of the coherent source defines the initial state and the signal to be sensed interacts with the spin. In a proof-of-principle experiment, we separate the state preparation from the interaction by applying the signals only during the sensing period for a time *τ*. Here, we use the fact that we generate the signal with an arbitrary waveform generator, giving us full control, see Methods 4.1. As described above, we will now perform a series of measurements that would allow us to coherently measure the phase of the signal at the start of each measurement. Because the spectral resolution in heterodyne measurements is given by the reference and does not depend on the measurement time by (1/*τ*), it allows us to sense a multimode signal in parallel, illustrated as a second signal in the lower panel of Fig. 2a analogous to a lock-in amplifier^24^. In the experiment, we measured a coherent two-frequency MW signal with frequencies *ω*_1_ = 2*π* ⋅ (4139.4 + 5 ⋅ 10^−2^) MHz, and *ω*_2_ = 2*π* ⋅ (4139.4 + 5 ⋅ 10^−2^ + 5 ⋅ 10^−4^) MHz. With respect to the reference frequency *ω*_ref_ = 2*π* ⋅ 4139.4 MHz, they differ by Δ*ω*_1_ = 2*π* ⋅ 50.138 kHz and Δ*ω*_2_ = 2*π* ⋅ 50.620 kHz respectively, slightly adjusted to have an integer number of cycles within one AWG sequence length (see Methods 4.2) . Both signals have equal amplitudes of Ω_1_ = Ω_2_ = 2*π* ⋅ 3.6 MHz and are applied for 34.2 ns. As the field amplitudes of the reference and the signal are much larger than the ${T}_{2}^{* }=50\, \upmu {\rm{s}}$ limit of the spin transition, they interact resonantly with the spin. When we perform a series of 10^6^ measurements, we record on average $\overline{n}=0.14$ photons per measurement. We extract the demodulation signal from the autocorrelation, as defined in Eq. (7) and shown in Fig. 2b. In the autocorrelation, we clearly see the beating of the two signals and the enlarged view in Fig. 2c shows the sinusoidal oscillations expected from the theoretical derivation above. We demonstrate the high spectral resolution by taking the fast Fourier transform (FFT) of the autocorrelation signal, as shown in Fig. 2d. We observe a splitting of the two peaks, corresponding to the frequency difference of (*ω*_1_ − *ω*_2_ = 2*π* ⋅ 482 Hz). The narrow linewidth of just 1 Hz and the measured frequency difference of 500 Hz demonstrate the spectral resolution beyond the coherence time of the sensor, which is typically in the range of 1/*T*_2_ > kHz.

Furthermore, we analyze the peak width in Fig. 2e by computing the correlation function up to the correlation length *N* and extract the linewidth of its spectral peak with a Lorentzian fit. For measurement of Δ*ω* = 2*π* ⋅ 75 kHz, *τ* = 1.824 μs, *T* = 4.505 μs and Ω_0_ = 2*π* ⋅ 111 kHz, we extract a 300 mHz linewidth for a correlation length of 3 seconds. We find that the linewidth is Fourier-limited, equal to the inverse of the maximal computed correlation length (1/(*N* ⋅ *T*) scaling). The achieved linewidth is a 3 orders of magnitude improvement compared to the 1/*T*_1_ ≈ 0.5 kHz lifetime limit. This would imply that we are now sensitive to sub ppb (parts per billion) changes in the frequency of applied fields. While this demonstrates the high spectral resolution for MW-sensing, we investigate the control of the interaction of the spin with the MW field below.

### Dynamical decoupling and sensitivity

**Fig. 3:**
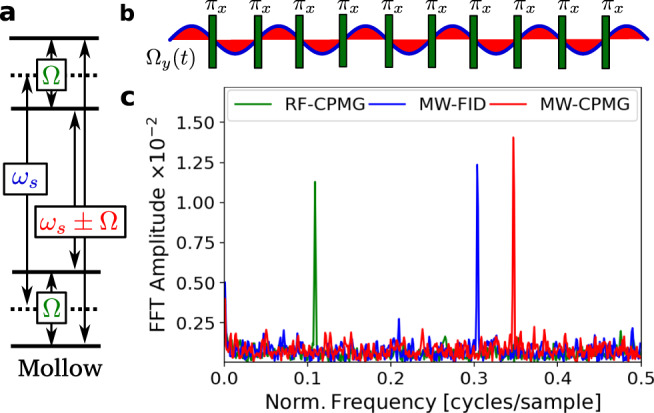
Heterodyne detection is compatible with dynamical decoupling sequences. **a** A decoupling sequence creates from the initial two-level system, with transition *ω*_*s*_, a Mollow triplet with two detection sidebands at *ω*_*s*_ ± Ω_dd_, where ${{{\Omega }}}_{{\rm{dd}}}=\frac{\pi }{{\tau }_{{\rm{dd}}}}$, and a low frequency transition Ω_dd_. A MW field resonant to such a sideband is rotating in the spin’s reference frame. **b** The phase of the oscillating field Ω_*y*_ (*t*) is sensed relative to the pulse sequence, here CPMG (*π*_*x*_-pulses). **c** Demodulated signal spectra of three heterodyne measurements, each with one Mollow triplet transitions. Blue: resonant transition *ω*_*s*_, green: RF-frequency Ω_dd_ and red: Mollow sideband *ω*_*s*_ + Ω_dd_. For comparison, we normalized the frequency axis to the sampling rate 1/*T* with *T*_RF-CPMG_ = 23.094518 μs, *T*_MW-FID_ = 4.505507 μs, and *T*_MW-CPMG_ = 70.681507 μs.

In the discussion above, we applied the signal only during the sensing time. Now we introduce the pulsed Mollow triplet, created by dynamical decoupling (DD), which effectively gives control over the interaction with the signal. Such decoupling sequences create a new dressed basis with new energy eigenstates, as shown in Fig. 3a. While in most sensing applications of decoupling sequences, the low frequency transition given by the strength of the driving Ω_dd_, is studied, we aim at sensing microwave fields and hence consider the Mollow sidebands at the absorption frequencies *ω*_*s*_ ± Ω_dd_, described in references^8,25^. This allows us to control the interaction of the sensor with the signal field. Additionally during the decoupling sequence, the sensor lifetime increases up to *T*_1*ρ*_, which is by orders of magnitude larger than ${T}_{2}^{* }$^26^. It is essential to have a precise reference frequency, which is achieved best when Ω_dd_ is independent of the power of the dressing fields. Here, we analyze a pulsed Mollow scheme which creates sidebands at Ω_dd_ = *π*/*τ*_dd_, with *τ*_dd_ as the inter-pulse spacing. This scheme is robust to power fluctuations (i.e., fluctuations in Ω_dd_), which typically challenges continuous-wave Mollow methods^8^. The interaction of the pulse train with the MW signal field is schematically drawn in Fig. 3b. The *π*-pulses construct an integration of the rotating component of the MW signal field. It becomes apparent that the phase of the oscillations relative to the pulse train alters the spin evolution. It is this effect that makes it suitable for heterodyne detection. The integration leads to a maximal sensor phase pickup of 2Ω_0_*τ*/*π*, with *τ* being the total sensing time (see Supplementary Eq. 13). The sensor phase pickup leads to a rotation of the initial state out of the x-y-plane towards the poles, analogous to Rabi oscillations. Experimentally, we modified the scheme of Fig. 2a, where we now apply the pulse train during the sensing time. In Fig. 3c, we show the spectrum of the measurement autocorrelation with frequency axis normalized to the sampling frequency 1/T. We applied the Carr-Purcell-Meiboom-Gill-Sequence (CPMG) as a dynamical decoupling sequence. For the heterodyne scheme, we used 10 repetitions, leading to a total sensing time of 68 μs. We applied a microwave signal of Δ*ω* = 2*π* ⋅ 79.96680000 kHz. The starting phase at each measurement gives the demodulation frequency by $\delta \omega =({{\Delta }}\omega T){\rm{mod}}(2\pi )/T$. In our experimental demonstration of pulsed Mollow heterodyne sensing, the demodulation frequency is larger than the Nyquist frequency, finally resulting in $\delta \omega =\left(2\pi -({{\Delta }}\omega T){\rm{mod}}(2\pi )\right)/T$. This gives an expected demodulation frequency of *δω* ≈ −2*π* ⋅ 4.921033 kHz, with *T* = 70.681507246 μs. Experimentally, we observed the demodulation frequency at *δω* ≈ 2*π* ⋅ 4.921027(14) kHz. The demodulation peak becomes 0.3448 after normalization with the sampling rate 1/*T*, as shown in Fig. 3c. We observe a Fourier-limited linewidth by the correlation length *NT* (1/(*NT*) scaling) for the Mollow peak (see Supplementary Fig. 2). As a comparison, we also show the low frequency transition Ω_*dd*_, the well-studied heterodyne peak for NMR and RF detection, and the resonant absorption described in the discussion of Fig. 2e. We see that all transitions in the dressed level scheme can be sensed in our heterodyne scheme and therefore relative to an external reference.

With dynamical decoupling, the lifetime of the spin increases from ${T}_{2}^{* }$, for a free induction decay, to *T*_1,*ρ*_, for the dressed rotating frame. This results in an increased sensitivity^24^. We estimated the sensitivity for our protocol to $\eta =(203\pm 15)\ {\rm{nT}}/\sqrt{{\rm{Hz}}}$ from the analysis of the signal-to-noise ratio in the power spectral density (see Methods 4.3). We further compare the extracted sensitivity to a shot-noise limited NV sensitivity, leading to $141\ {\rm{nT}}/\sqrt{{\rm{Hz}}}$. The reduction by a factor of $\sqrt{2}$ originates in our signal response scaling with ${{{\Omega }}}_{0}\tau /\sqrt{2}$ (see Eq. (7)). We project the sensitivity of heterodyne MW detection with NV centers to $26\ {\rm{nT}}/\sqrt{{\rm{Hz}}}$ for optimal experimental parameters, comparable to common sensitivities of NV sensing^27^.

### Tuning of interactions with Floquet states

**Fig. 4:**
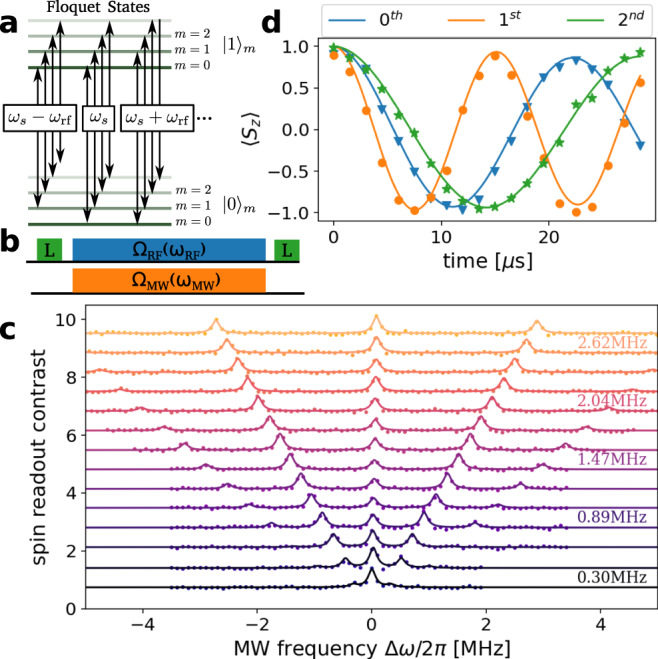
MW detection with a longitudinal RF dressing. **a** The RF driving creates the new energy states *E*_0,*m*_ = *mω*_rf_ and *E*_1,*m*_ = *ω*_*s*_ + *mω*_rf_. Generating sidebands with *ω* = *ω*_*s*_ + Δ*mω*_rf_. **b** Experimental scheme for probing these transitions. Initialization and readout with green laser pulses, followed by simultaneous RF (blue) and MW (orange) driving. **c** Optically detected magnetic resonance spectrum with varied *ω*_rf_ showing the Floquet sidebands at multiples of *ω*_rf_. **d** Rabi oscillations at the central peak (0th), first, and second sideband. The amplitude of RF is chosen such that the Rabi frequency is largest for the first sideband and is described with ${\nu }_{{\rm{Rabi,n}}}={J}_{n}(\frac{2.5\, {\rm{MHz}}}{1.45\, {\rm{MHz}}})\cdot 0.125\,{\rm{MHz}}$.

In the above, we have only considered control over the sensor using MW manipulation. In the following, we further extend this by dressing the sensor states with a strong longitudinal RF-drive. The advantage is, that we naturally create sidebands depending on the frequency of the RF field, hence making it robust to amplitude fluctuations without the requirement of a pulsed operation and more importantly the power requirements for the MW channel relax, which becomes particularly relevant for high MW frequencies. While RF-control over the two-level system has been studied^20–22^, we use it here as a resource for heterodyne detection. The Hamiltonian under such RF field driving with frequency *ω*_rf_ and strength Ω_rf_ becomes:

8 $$H=\frac{{\omega }_{s}}{2}{\sigma }_{z}+{{{\Omega }}}_{{\rm{rf}}}\cos ({\omega }_{{\rm{rf}}}t){\sigma }_{z}+{{{\Omega }}}_{0}\sin (\omega t+{\phi }_{0}){\sigma }_{x},$$

where we consider a strong driving Ω_rf_ ⪅ *ω*_rf_. We omit the terms transverse to the NV-axis, which do not contribute significantly to the Floquet dynamics, but mainly cause a Ramsey-Bloch-Siegert frequency shift^28^ analogous to a change in *ω*_*s*_ (see Supplementary Note 4 for details). The strong RF-drive results in the new energy levels depicted in Fig. 4a. The state $\left|0\right\rangle$ becomes $\left|0,m\right\rangle$, where *m* denotes a new quantization index or the energy eigenvalues of *E*_0,*m*_ = *mω*_rf_ and *E*_1,*m*_ = *ω*_*s*_ + *mω*_rf_, respectively. These states allow transitions with frequency *ω*_*s*_ ± Δ*m* *ω*_rf_, where Δ*m* denotes the change in the additional energy level index for the transition between $\left|0\right\rangle$ to $\left|1\right\rangle$, see Fig. 4a. Because of the energy splitting given by the frequency of the RF-drive, it becomes clear that these sidebands are ideal candidates for heterodyne detection. The transition strength of the sidebands is given by ${P}_{{{\Delta }}m}={J}_{{{\Delta }}m}\left({{{\Omega }}}_{{\rm{rf}}}/{\omega }_{{\rm{rf}}}\right)$, with *J*_Δ*m*_ Bessel functions of the first kind of order Δ*m*. Hence, one could tune the transition strength by adjusting *x* = Ω_rf_/*ω*_rf_ to be at a local maximum. At this local maximum, we have a quadratic dependence of the transition strength on power fluctuations ${({{\Delta }}{\Omega }_{{\rm{rf}}})}^{2}$, which underlines the robustness of the method. As shown in Fig. 4b, we experimentally probe these new transition frequencies by initializing the sensor spin with a green laser pulse, followed by a simultaneously applied strong RF field and a weak MW-probe field. The state is finally readout with another laser pulse. The optically detected magnetic resonance (ODMR) of the RF-dressed states is shown in Fig. 4 where we measure the spin readout contrast using single shot readout^29,30^, with changing MW-probe frequency for RF-driving frequencies between 0.3–2.9 MHz illustrated by a vertical offset. Finally, we picked the driving frequency of 1.45 MHz and performed Rabi oscillations on the central peak (0th), first, and second sideband, to confirm the dependence on the transition strengths *J*_Δ*m*_(*x*) ⋅ Ω_Rabi_, where Ω_Rabi_/2*π* = 125 kHz. We measured Ω_Rabi,0_(*x*)/(2*π*) = 45 kHz, Ω_Rabi,1_/(2*π*) = 66 kHz, and Ω_Rabi,2_/(2*π*) = 35 kHz, resulting into a value of *x* = 1.72, which is close to the maximum of *J*_1_(*x*).

**Fig. 5:**
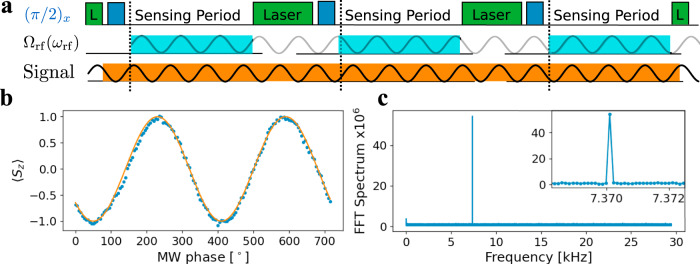
Heterodyne detection using Floquet dressed states. **a** Experimental scheme: Optical initialization and readout of the electron spin (green), preparation of the rotating frame by a *π*/2 pulse (blue). In addition, a RF field is applied for the sensing period (light blue), which is coherent between measurements. The demodulation frequency between a MW signal (orange) and the reference is given by Δ*ω* − *ω*_rf_ for the first sideband. **b** Sensor response as a function of the signal phase in the Floquet dressed system, while the RF phase is fixed. **c** Heterodyne detection of the MW-field in a series of 10^6^ coherent measurements in the Floquet dressed system. The phase of the RF field is modulated by *ϕ*_*i*+1_ = *ϕ*_*i*_ + 45°, giving maximal sensor response (Δ*ω* = *ω*_rf_) while moving the signal frequency to *ν*_demod._ = 1/8 of the sampling frequency.

After introducing the new dressed states, we show that they are suitable for heterodyne detection (see Supplementary Note 4). The RF-driving results in additional oscillations of the spin in the rotating frame around the *z*-axis and therefore can be interpreted as phase gates. Similar to the pulsed Mollow absorption shown in Fig. 3b, we have z-rotations instead of x-rotations, that follow the frequency of the RF-drive. Depending on the phase of the RF drive, the timing of these gates changes and creates a phase-sensitive sequence. Higher harmonics appear due to saturation of the phase gates, surpassing 2*π* rotation within one period, creating higher-order frequency acceptance. Experimentally, we show heterodyne detection in the RF-dressed basis by the scheme shown in Fig. 5a. We prepare the initial state of NV electron spin with a laser pulse and a *π*/2 pulse from a coherent source, yielding a $\left|{\psi }_{{\rm{init}}}({\phi }_{{\rm{ref}}})\right\rangle$ state. In addition, we apply strong RF drive during the sensing period. The signal interferes with the spin only during the sensing period, giving us control over the interaction and we expect a demodulated frequency of *ω*_signal_ − Δ*m* *ω*_rf_ − *ω*_*s*_. We experimentally show the phase sensitivity of our sensing block, in Fig. 5b by measuring its response to varying initial phases of the MW signal. We observe clean sinusoidal oscillations, clearly illustrating the phase sensitivity. Finally, we perform sequential measurements of a MW-field and plot the Fourier spectrum of the autocorrelation. The experimental conditions were adjusted such that *ω*_signal_ − Δ*m* *ω*_rf_ − *ω*_*s*_ = 0. The experimental settings were *ω*/(2*π*) = 4140.664 MHz, *ω*_*rf*_/(2*π*) = 1.45 MHz, *ω*_*s*_/(2*π*) = 4139.214 MHz, *τ* = 5 μs, *T* = 16.960 μs and Ω/(2*π*) = 125 kHz. We varied the phase of the RF dressing field depending on the measurement index as *ϕ*_*n*+1_ = *ϕ*_*n*_ + 45^∘^, moving the demodulation frequency to 1/8 of the sampling frequency. Therefore, we observe the correlation spectrum peak at 1/8 of the sampling frequency at 7.370 kHz. The peak width is Fourier-limited (see Supplementary Fig. 2).

## Discussion

This work applies heterodyne quantum sensing techniques to GHz frequencies, extending previous protocols^15,16^. At the same time, it converts absorption-based MW sensing to a heterodyne scheme, extending the resolution beyond *T*_1_ of the sensor^8,10^, i.e. below 1 Hz. The heterodyne detection protocol presented in this work can be applied to three different systems, i.e., a two-level system, a Mollow triplet, and a Floquet dressed system with the key requirement being the ability to make them sensitive to the phase of the incident signal. While the simplest case of a two-level system shows the working principle, the addition of Floquet RF driving to heterodyne sensing allows us to control the interaction with the signal and create stable sidebands that are independent of the frequency band of the two-level system. Furthermore, the Mollow dressing improves the sensitivity from ${T}_{2}^{* }$ to *T*_1,*ρ*_ limit, thus leading to estimated sensitivities of $203\ {\rm{nT}}/\sqrt{{\rm{Hz}}}$ for our single NV centers and a projected sensitivity of about $26\ {\rm{nT}}/\sqrt{{\rm{Hz}}}$ for a measurement with optimized sensitivity. In this work, we overcome the spectral resolution problem for quantum sensors in the microwave regime. This is important when sensing weak and highly coherent MW signals, for example in Masers^31^, Quantum Radar^2,3^ and Doppler velocimetry technologies^32^, weak cosmic radiation^1^ or wireless communication protocols. Our approach can be applicable to a broad range of *B*_0_ field where NV spin manipulations are possible, which allows to sense MW frequencies in the range ≈0–100 GHz^9,33^. Furthermore, the pulsed Mollow heterodyne sequences can be straightforwardly applied to high dynamic range sensing of large signal MW fields, as already demonstrated for RF fields^34^. In addition, the heterodyne approach in sensing leads to the concept of sequential weak measurements of quantum systems, which potentially is important in measuring the quantum behavior of mesoscopic bosonic or fermionic systems at high frequencies and for quantum feedback^35^. The heterodyne sensing protocols presented here could be also applied to other qubit systems, such as transmon qubits^4–6^ that naturally operate in the MW regime^36^. Recently, such MW heterodyne sensing was applied in Rydberg atoms to sense electric MW fields^32^. Furthermore, heterodyne sensing protocols for NV centers and other quantum sensors were, independently from this study, proposed and studied in reference^37^.

## Methods

### Experimental setup

**Fig. 6:**
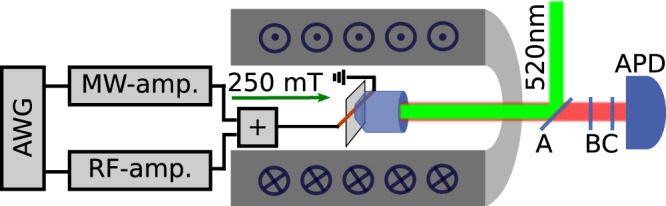
Single NV-center confocal setup with microwave control. The diamond with individual NV-centers is studied with a confocal microscope in the center of a room temperature bore of a superconducting magnet, operated at 250 mT. For excitation, a 520 nm diode laser is used, the beam is reflected from a wedged mirror (A) and coupled into the objective. The fluorescence of the NV is passing again through the wedged mirror (A), through a pinhole 50 μm, (B) and finally through a long-pass filter, 650 nm, (C) before getting detected with an avalanche photon detector (APD) (Perkin–Elmer SPCM). The microwave and radio frequencies are generated on a two-channel arbitrary waveform generator, sent to amplifiers, and combined before the microwave structure, where the diamond is glued on.

The scheme of the experimental setup is shown in Fig. 6. The diamond crystal is positioned within a room temperature bore of a superconducting NMR-magnet (Scientific Magnetics). The magnetic field was about 250 mT and aligned with NV center quantization (Z) axis, leading to a transition frequency of 4139.3 MHz between $\left|0\right\rangle$ and $\left|-1\right\rangle$ for the nuclear spin projection *m*_*I*_ = +1. For the optical excitation and collection of the fluorescence of the NV center, we use an immersion oil objective with a numerical aperture NA = 1.35 and detect it with an avalanche photo-diode (APD) (Perkin–Elmer SPCM), capable of detecting single photons. A 520 nm diode laser is used for excitation, which can be directly turned on within 10 ns. The NV microwave transitions and RF manipulation are generated on a Keysight 8190A arbitrary waveform (AWG) with 12 GSamples/s. The same device controls the laser diode switching and the data acquisition triggering. We amplify the first channel (MW) up to about 40 dBm power with Hughes-Traveling Wave Tube 8010H amplifier (TWT, max. 7 MHz Rabi frequency), the second channel (RF) to about 52 dbm with RF amplifier (Amplifier Research 150A250) leading to an oscillating field of 0.1 mT along the NV quantization axis. For the experiments on heterodyne detection with pulsed Mollow absorption, we changed the first channel TWT amplifier to a solid state amplifier (Mini-Circuits ZHL-42, max. 0.4 MHz Rabi frequency). We combine the MW and RF channels before coupling to a coplanar waveguide, therefore the two channel fields have the same spatial distribution. The diamond sample used is a 2 mm × 2 mm × 80 μm, (111)-oriented polished slice from a ^12^C-enriched (99.995%) diamond crystal. The crystal was grown by the temperature gradient method under high-pressure high-temperature conditions at 5.5 GPa and 1350 ^∘^C, using high-purity Fe-Co-Ti solvent and high-purity ^12^C-enriched solid carbon. The single NV centers were created from intrinsic nitrogen by irradiation with 2 MeV electrons at room temperature with a total fluence of 1.3 ⋅ 10^11^ cm^2^ and annealed at 1000 ^∘^C (for 2 h in vacuum). The typical lifetimes for the NV centers in this slice are ${T}_{2}^{* }=50\ \upmu {\mathrm{s}}$ and *T*_2_ ≈ 300 μs^23^.

### Data processing

We record our data with a Swabian Instruments time tagger giving us the photon counts gated by one of the AWG synchronization markers, which also controls the readout laser pulse. This leads to a waveform data set with a sampling time given by the sequence duration of the AWG. Because of the confocal setup, we refocus the confocal spot every 10^6^ measurements, which is approximately a few seconds to a minute, depending on the sequence duration, and is the maximum time we are performing coherent measurements. The MW signals were generated using AWG. The AWG memory is enough to build a sequence of up to 100 measurements. This allows to adjust the sequence length such that all frequencies used within the sequence have integer number of cycles within the total sequence duration. As a result, by repetition, the AWG could create phase coherent frequency signals on the timescale larger than allowed by memory constraints. We detect on average 0.14 photons and have a typical contrast of 29%. To extract the signal, we perform an autocorrelation of the photon time trace. We normalize the correlated time trace *C* (*n*) by the number of data points of each correlation length (e.g., 10^6^ − *n*), leading to a constant amplitude of a sinusoidal signal across all correlation lengths. We average multiple experiments after autocorrelation. In our demonstration experiment, the signals are generated from a coherent single source, hence the extracted oscillations are non-decaying leading, to a Fourier-limited linewidth (1 point in Fourier space).

### Sensitivity estimation

The sensitivity is retrieved from the analysis of series of 10^6^ measurements, resulting in a string of photon counts, with a sampling interval *T* = 70.6815 μs. We determine a signal-to-noise ratio of 134 ± 19 in the power spectral density (PSD) of the photon counts, equal to the Fourier transform of the autocorrelation. The applied signal field was 660 nT, equivalent to a maximal phase ${{{\Phi }}}_{\exp }=0.57\pi$ close to the maximum response of the sensor (see Supplementary Eq. (13)). In addition, we characterized the signal strength and field from an absorption measurement (see Supplementary Fig. 1). From there, we estimate the minimum detectable sensor phase value as ${{{\Phi }}}_{\min }=(0.0293\pm 0.0015)\ \pi$. This translates to a minimal oscillating magnetic field (twice as large because of the RWA) of ${B}_{\min }=(2\pi {{{\Phi }}}_{\min })/(2\gamma \tau )=(24\pm 2)$ nT. Our total measurement time was 71 seconds. Hence, we conclude a sensitivity of $\eta =(203\pm 15)\ {\rm{nT}}/\sqrt{{\rm{Hz}}}$. To quantify the extracted sensitivity, we compare it to the theoretical sensitivity estimation for shot-noise limited AC sensing, which results in $141\ {\rm{nT}}/\sqrt{{\rm{Hz}}}$ (see Supplementary Eq. (36)). The theoretical value differs from the extracted value by $\sqrt{2}$, because we analyze our signal in the PSD. The signal amplitude in the PSD is given by $M\frac{1}{2}{{{\Phi }}}_{{\rm{sensor}}}^{2}$ (see Eq. (7)), which is a factor of $\sqrt{2}$ reduced compared to protocols with a linear response. For optimized sensor parameters, in particular full contrast (0.08 → 0.3) and maximal sensing time (68 μs → 300 μs), we project the sensitivity of this protocol to 26 ${\rm{nT}}/\sqrt{{\rm{Hz}}}$.

## Supplementary information

Supplementary Information

## Data Availability

Data supporting the findings of this study are available within the article and its Supplementary Information and from the corresponding authors upon request.

## Code availability

Custom computer code (python) used in the theoretical studies and experimental analysis are available from the corresponding authors upon reasonable request.
