# Supplementary material for: Heterodyne sensing of microwaves with a quantum sensor
Source: Nat Commun. 2021 May 12;12:2737. doi: 10.1038/s41467-021-22714-y (PMC8114934; doi:10.1038/s41467-021-22714-y)
Supplement: Supplementary file 1 — Supplementary Information [file 41467_2021_22714_MOESM1_ESM.pdf]

Supplementary Information: Heterodyne Sensing of Microwaves with a Quantum Sensor

Jonas Meinel, [1,2*] Vadim Vorobyov, [1*] Boris Yavkin,[1] Durga Dasari, [1,2]
Hitoshi Sumiya,[3] Shinobu Onoda,[4] Junichi Isoya,[5] Jörg Wrachtrup [1,2*]

[1] 3rd Institute of Physics, University of Stuttgart and
Institute for Quantum Science and Technology IQST, 70569 Stuttgart, Germany
[2]Max-Planck Institute for Solid State Research, Stuttgart 70569, Germany
[3]Advanced Materials Laboratory, Sumitomo Electric Industries Ltd., Itami 664-0016, Japan
[4]Takasaki Advanced Radiation Research Institute, National Institutes
for Quantum and Radiological Science and Technology, Takasaki 370-1292, Japan
[5] Faculty of Pure and Applied Sciences, University of Tsukuba, Tsukuba 305-8573, Japan.
Correspondence: * j.meinel@pi3.uni-stuttgart.de, v.vorobyov@pi3.uni-stuttgart.de,
j.wrachtrup@pi3.uni-stuttgart.de

Here we show (i) the theoretical derivations of the heterodyne sensing, with optional dressing fields, (ii) the sensitivity estimation for a shot noise limited sensor, (iii) the linewidth over the correlation length for the datasets shown in Figure 3 and 5 in the main text leading to the Fourier limited linewidth. Finally we show a setup description of the MW field and sample orientation.

Supplementary Note 1: Evolution under phase dependent Hamiltonian

The essence of our work is the phase dependent measurement outcome. In the following we derive this outcome from the protocol shown in the main text Figure 2. We consider the following

phase dependent Hamiltonian :

$$H' = \frac{\Delta\omega}{2}\sigma_z + \frac{\Omega_0}{2}\cos(\phi_0)\sigma_x + \frac{\Omega_0}{2}\sin(\phi_0)\sigma_y, \tag{1}$$

where $\Delta\omega$ is given by $\Delta\omega = \omega_s - \omega$. When we create the time evolution operator U we get:

$$\begin{aligned} U =& \exp(-iH't) \\ =& \exp\left(-\frac{i}{2}\Omega' t(\mathbf{n}\cdot\mathbf{\Sigma})\right) \\ =& \cos(\Omega' t)\mathbb{1} - i\frac{\sin(\Omega' t)}{\Omega'} \\ & \times \left(\Omega_0\cos(\phi_0)\sigma_x + \Omega_0\sin(\phi_0)\sigma_y + \Delta\omega\sigma_z\right), \end{aligned} \tag{2}$$

where the $\mathbf{\Sigma} = (\sigma_x, \sigma_y, \sigma_z)$ is the Pauli vector, $\mathbf{n}$ is the normalized field vector and $\Omega' = \sqrt{\Omega_0^2 + \Delta\omega^2}$ is the generalized Rabi frequency. We assume an initial state of $|+\rangle = \frac{1}{\sqrt{2}}(|0\rangle + |1\rangle)$, leading to the following state after evolution:

$$\begin{aligned} |\psi(t)\rangle = & \left(\cos(\Omega' t) - i\frac{\sin(\Omega' t)}{\Omega'}\Omega_x\right)|+\rangle \\ & + \left(\frac{\sin(\Omega' t)}{\Omega'}\Omega_y - i\frac{\sin(\Omega' t)}{\Omega'}\Delta\omega\right)|-\rangle, \end{aligned} \tag{3}$$

where $\Omega_x = \Omega\cos\phi_0$, $\Omega_y = \Omega\sin\phi_0$. Finally we take the expectation value of S_z, equal to our measurement outcome, given by:

$$\begin{aligned} \langle S_z\rangle =& \sin(2\Omega' t)\frac{\Omega_0}{2\Omega'}\sin(\phi_0) + \sin^2(\Omega' t)\frac{\Omega_0\Delta\omega}{\Omega'^2}\cos(\phi_0) \\ \approx& \Omega_0 t\sin(\phi_0), \end{aligned} \tag{4}$$

for small phase acquisition ($2\Omega' t \ll 1$).

Supplementary Note 2: Analytical Solution for Mollow Dressed System

For control of the interaction with the signal field and long coherence times we apply dressed states, the Mollow triplet. The Hamiltonian in the lab frame is given by:

$$H = \frac{\omega_s}{2}\sigma_z + \Omega_{\text{dressing}}\sigma_x \cos(\omega_1 t) + \gamma\Omega_0\sigma_x \cos(\omega t + \phi), \tag{5}$$

with Ω_{dressing} the strength and ω_1 the frequency of the continuous dressing field. When we go into the rotating frame of the fast oscillations ω_1, assuming $\omega_1 = \omega_s$, we get:

$$H' = \left(\frac{\Omega_{\text{dressing}}}{2} + \frac{\gamma\Omega_0}{2}\cos(\Delta\omega t + \phi)\right)\sigma_x + \frac{\gamma\Omega_0}{2}\sin(\Delta\omega t + \phi)\sigma_y, \tag{6}$$

ignoring the term $2\,\omega_1 \gg \Omega_{\text{dressing}}$ (Rotating Wave Approximation (RWA)). Finally we can go into the second rotating frame:

$$H'' = \Delta''\sigma_x + \frac{\gamma\Omega_0}{4}(\cos\phi\,\sigma_z - \sin\phi\,\sigma_y), \tag{7}$$

where $\Delta'' = \frac{1}{2}(\Omega_{\text{dressing}} - \Delta\omega + \gamma\Omega_0\cos(\Delta\omega t + \phi)) \approx \frac{1}{2}(\Omega_{\text{dressing}} - \Delta\omega)$ and we assumed $2\Delta\omega \gg \Delta''$.

Supplementary Note 3: Analytical Pulsed Mollow Sensing

The pulsed Mollow absorption can be understood as a dynamical decoupling sequence for sensing fields along the x,y-axis, instead of the z-axis of the quantum sensor.[1,2] We start with the Hamiltonian H' in the rotating frame of the decoupling pulses, which we assume perfect π pulses ($\omega_{\text{dd}} = \omega_s$):

$$H' = \frac{\Omega}{2}\cos((\omega - \omega_{\text{dd}})t + \phi_0)\,\sigma_x + \frac{\Omega}{2}\sin((\omega - \omega_{\text{dd}})t + \phi_0)\,\sigma_y, \tag{8}$$

when we integrate the Hamiltonian, we get the evolution operator U as:

$$U(H, t_j) = \exp\left(-i(I_x(t_j)\sigma_x + I_y(t_j)\sigma_y)\right), \tag{9}$$

where I_x, I_y are the solutions of the integral starting from t_j until $t_j + \tau_{\mathrm{dd}}$, and τ_{dd} is the inter pulse spacing. In our paper we use the CPMG sequence which is a series of identical π-pulses spaced with the time τ_{dd} and repeated for even number M. We assume resonance of the dd-pulses ($\omega_s = \omega_{\mathrm{dd}}$) and therefore the pulses become σ_x. The evolution operator under this sequence becomes:

$$U_{\mathrm{dd}} = \prod_{n=0}^{M/2} U(H', t_{2n})\sigma_x U(H', t_{2n+1})\sigma_x. \tag{10}$$

Now we only have to use the commutators of the Pauli matrices to find $\sigma_x U(H', t_j)\sigma_x$ using the relation $\sigma_x \exp(A)\sigma_x = \exp(\sigma_x A \sigma_x)$, leading to:

$$\sigma_x U(H', t_j)\sigma_x = \exp\left(-i(I_x(t_j)\sigma_x - I_y(t_j)\sigma_y)\right), \tag{11}$$

note the now changed minus sign for the σ_y term, while σ_x remains unchanged. This leads to the rectangular $[+1, -1]$ filter function spaced with τ_{dd} when we calculate the overall evolution which eventually becomes:

$$U_{\mathrm{dd}} = \prod_{n=0}^{M} \exp\left(-i(I_x(t_n)\sigma_x + (-1)^n I_y(t_n)\sigma_y)\right). \tag{12}$$

We further include the modulation in the integral I_y using rect the rectangular function leads to the final response function of:

$$\begin{aligned} I'_y &= \frac{\Omega}{2}\int_0^{\tau} \mathrm{dt}\left(1 - 2\,\mathrm{rect}\left(\sin\left(\frac{\pi}{\tau_{\mathrm{dd}}}\mathrm{t} + \frac{\pi}{4}\right)\right)\right)\sin((\omega - \omega_{\mathrm{dd}})\mathrm{t} + \phi_0), \\ &\approx \frac{2\Omega\tau}{\pi}\frac{\sin\left(\left(\frac{\pi}{\tau_{\mathrm{dd}}} - \omega\right)\tau/2\right)}{\left(\frac{\pi}{\tau_{\mathrm{dd}}} - \omega\right)\tau/2}\sin\left(\phi + \left(\frac{\pi}{\tau_{\mathrm{dd}}} - \omega\right)\tau/2\right). \end{aligned} \tag{13}$$

while $I'_x \approx 0$ for large integration time. In opposite to the other protocols the pulsed Mollow absorption differs in the sensor response. While for the description in the main text the phase

information is encoded in the direction of the effective field, for pulsed Mollow sensing it is encoded in the sensor phase pickup. The sensor response is given by:

$$\langle S_z \rangle = \sin(I'_y) = \sin\left(\frac{2\Omega\tau}{\pi}\sin(\phi_0)\right), \tag{14}$$

where we assumed $(\omega - \omega_{\rm dd})/(2\pi) = 1/(2\tau_{\rm dd})$. This leads to the possibility of high dynamic range sensing.

Supplementary Note 4: Analytical Solution in the Strongly Driven System with Longitudinal RF field

The following was adapted from[3] to our situation. We start with the Hamiltonian of the driving:

$$H = \frac{\omega_s}{2}\sigma_z + \frac{\Omega_{\rm rf}}{2}\cos(\omega_{\rm rf}t)\sigma_z + \Omega_1\cos(\omega_{\rm mw}t + \phi_0)\sigma_x, \tag{15}$$

where $\Omega_{\rm rf}$ is the strong RF longitudinal drive with frequency $\omega_{\rm rf}$. This Hamilitonian we transform in the rotating frame with the evolution operator:

$$U = \exp\left(\frac{i\sigma_z}{2}\left(\omega_s t + \frac{\Omega_{\rm rf}}{\omega_{\rm rf}}\sin(\omega_{\rm rf}t)\right)\right). \tag{16}$$

The Hamiltonian in the rotating frame becomes:

$$H' = \Omega_1\cos(\omega_{\rm mw}t + \phi_0)\, U^\dagger\sigma_x U, \tag{17}$$

where $U^\dagger\sigma_x U$ is given by:

$$\begin{pmatrix} 0 & \mathrm{e}^{-\mathrm{i}\left(\omega_{\rm s}\mathrm{t} + \frac{\Omega_{\rm rf}}{\omega_{\rm rf}}\sin(\omega_{\rm rf}\mathrm{t})\right)} \\ \mathrm{e}^{\mathrm{i}\left(\omega_{\rm s}\mathrm{t} + \frac{\Omega_{\rm rf}}{\omega_{\rm rf}}\sin(\omega_{\rm rf}\mathrm{t})\right)} & 0 \end{pmatrix}. \tag{18}$$

We can further use:

$$\exp\left(iz\sin(\omega t)\right) = \sum_{k=-\infty}^{\infty} J_k\left(z\right)\exp\left(k\omega t\right), \tag{19}$$

where J_k are the Bessel function of the first kind. This leads to the following matrix entries of $U^\dagger \sigma_x U$:

$$\begin{pmatrix} 0 & \sum J_k(\frac{\Omega_{\mathrm{rf}}}{\omega_{\mathrm{rf}}})e^{-i(\omega_s+k\omega_{\mathrm{rf}})t} \\ \sum J_k(\frac{\Omega_{\mathrm{rf}}}{\omega_{\mathrm{rf}}})e^{i(\omega_s+k\omega_{\mathrm{rf}})t} & 0 \end{pmatrix}. \tag{20}$$

Let's assume that the probe field amplitude is small $\Omega_1 \ll \omega_{\mathrm{rf}}$, which allows us to only look at one resonance and the probe field is close to resonance of a sideband $\omega_k = \omega_s + k\,\omega_{\mathrm{rf}}$. We get the effective Hamiltonian:

$$\begin{aligned} H'_k = &J_k\left(\frac{\Omega_{\mathrm{rf}}}{\omega_{\mathrm{rf}}}\right)\Omega_1\cos(\omega_{\mathrm{mw}}t+\phi_0) \\ &\times\left(\cos(\omega_k t)\sigma_x + \sin(\omega_k t)\sigma_y\right). \end{aligned} \tag{21}$$

Using the RWA, because of $\Omega_1 \ll \omega_{\mathrm{rf}}$, we further get:

$$H'_k = \frac{\Omega_k}{2}\left(\cos(\Delta' t+\phi_0)\sigma_x + \sin(\Delta' t+\phi_0)\sigma_y\right), \tag{22}$$

with $\Omega_k = J_k\left(\frac{\Omega_{\mathrm{rf}}}{\omega_{\mathrm{rf}}}\right)\Omega_1$ and $\Delta' = \omega_{\mathrm{mw}} - \omega_n$, which finally becomes:

$$H''_k = \Delta'\sigma_z + \frac{\Omega_k}{2}\left(\cos\phi_0\,\sigma_x + \sin\phi_0\,\sigma_y\right), \tag{23}$$

showing the dependants on the initial phase ϕ_0.

In our experimental realization we can not exclude a transverse component of the oscillating field. The Hamiltonian H_T including the transverse field is given by:

$$H_T = \frac{\omega_s}{2}\sigma_z + \cos(\omega_{\mathrm{rf}}t)\left(\frac{\Omega_{\mathrm{rf,x}}}{2}\sigma_x + \frac{\Omega_{\mathrm{rf,z}}}{2}\sigma_z\right) + \Omega_1\cos(\omega_{\mathrm{mw}}t+\phi_0)\sigma_x. \tag{24}$$

We solved the transition frequencies numerically and found an agreement with the reference[4] that transverse components are by orders of magnitude smaller and appear in doubled frequencies compared to longitudinal components. Additionally, the simulations showed a frequency shift, also known as Ramsey-Bloch-Siegert shift,[5,6] which changes all transition frequencies analogous to a change of the main magnetic field B_0. The findings of the numerical simulations

can be explained by simply studying the absolute of the effective magnetic field vector $\boldsymbol{\Omega}_{\text{eff}}$ for $\omega_s \gg \Omega_{\text{rf,x}}, \Omega_{\text{rf,z}}$:

$$\begin{aligned} |\Omega_{\text{eff}}| &= \sqrt{(\omega_s + \Omega_{\text{rf,z}} \cos(\omega_{\text{rf}} t))^2 + (\Omega_{\text{rf,x}} \cos(\omega_{\text{rf}} t))^2} \\ &= \omega_s + \frac{\Omega_{\text{rf,x}}^2}{4\omega_s} + \Omega_{\text{rf,z}} \cos(\omega_{\text{rf}} t) + \frac{\Omega_{\text{rf,x}}^2}{4\omega_s} \cos(2\omega_{\text{rf}} t) + O\left(\frac{\Omega_{\text{rf,x}}^2 \Omega_{\text{rf,z}}}{\omega_s^2}\right). \end{aligned} \tag{25}$$

The first perturbation term is the Ramsey-Bloch-Siegert shift given by $\frac{\Omega_{\text{rf,x}}^2}{4\omega_s} \approx 2\pi \cdot 25\,\text{kHz}$ assuming $\Omega_{\text{rf,x}} = 2\pi \cdot 20\,\text{MHz}$ and $\omega_s = 2\pi \cdot 4\,\text{GHz}$, the second is the Floquet dynamics under longitudinal drive as described in the main text and the third term are the double frequency Floquet dynamics strongly suppressed by about $\frac{\Omega_{\text{rf,x}}}{8\omega_s} \approx 0.625 \cdot 10^{-3}$. This leads to the simplifications in the main text, focussing on longitudinal Floquet dynamics.

Supplementary Note 5: Sensitivity Estimation

In the following we want to derive the sensitivity of the Mollow heterodyne sensing protocol. First, we estimate the sensor phase of the applied signal. We can not use Rabi oscillations on the Mollow sideband for the calibration of our protocol. Because our protocol is phase sensitive which would bias the result, see Supplementary Fig. 1a, where we show two measurements with dynamical decoupling leading to largely different effective Rabi frequencies. Instead, we selected two AWG amplitude 0.01 and 0.02 relative to 0.35 V and measured the sensor response when changing the starting phase of the signal. The measurement starts with a $\pi/2$ pulse, then followed by a CPMG-20 together with the MW signal and by another $\pi/2$ pulse. Finally, a readout laser pulse was applied. We selected the probe frequency and dynamical decoupling spacing to be $\Delta\omega = \frac{\pi}{\tau_{\text{dd}}} = 2\pi \cdot 75\,\text{kHz}$. The results are shown in Supplementary Fig 1b. The sensor response follows Supplementary Equation 13 as $\cos(\Phi_{\text{sensor}} \sin(\phi_{\text{signal}} + \phi_0))$ which we used as a fit function. We extracted a sensor phase pick-up of $\Phi_{\text{sensor},0.01} = 2.26 \pm 0.08$ radians for amplitude 0.01 and $\Phi_{\text{sensor},0.02} = 5.04 \pm 0.07$ for amplitude 0.02. For the heterodyne experiment we

moved the MW-signal away from the center frequency of the filter and used CPMG-10. This gives us a reduced phase pickup of $\Phi_{\mathrm{exp}} = \frac{1}{2}\Phi_{\mathrm{sensor},0.02} \cdot \mathrm{sinc}\left(\left(\frac{\pi}{\tau_{\mathrm{dd}}} - \Delta\omega\right)\tau/2\right) = 0.57\pi$ for $\tau_{\mathrm{dd}} = 6.8\,\mu\mathrm{s}$, $\tau = 68\,\mu\mathrm{s}$ and $\Delta\omega = 2\pi \cdot 80\,\mathrm{kHz}$. The sensor response is given by $S(\Phi_{\mathrm{exp}}) = J_1(0.57\pi) = 1.07$.

From single measurement series of 10^6 measurements we estimate the signal-to-noise ratio to $SNR_{\mathrm{psd}} = 134 \pm 19$, where the uncertainty was extracted from the standard deviation of 9 measurement series. This leads to the minimal sensor phase:

$$\Phi_{\mathrm{min}} = S^{-1}\left(\frac{S(\Phi_{\mathrm{exp}})}{\sqrt{SNR_{\mathrm{psd}}}}\right) \tag{26}$$

$$= \arcsin\left(\frac{1.07}{\sqrt{134}}\right) = (0.0293 \pm 0.0015)\ \pi, \tag{27}$$

$$\tag{28}$$

which results int he following minimal magnetic field:

$$B_{\mathrm{min}} = 2\frac{\pi}{2}\frac{\Phi_{\mathrm{min}}}{\gamma\tau} = 24 \pm 2\,\mathrm{nT} \tag{29}$$

$$\eta = B_{\mathrm{min}} \cdot \sqrt{MT} = (203 \pm 15)\,\mathrm{nT}/\sqrt{\mathrm{Hz}}, \tag{30}$$

with the total sensing time $\tau = 68\,\mu\mathrm{s}$, number of experiments $M = 10^6$ and repetition time $T = 71\,\mu\mathrm{s}$. The minimal magnetic field B_{min} is a factor of π larger because of the RWA (2) and the reduced sensor response in pulsed Mollow absorption ($\pi/2$, see Supplementary Equation 13).

In the following we compare the estimated sensitivity from the analysis of a single photon time trace to a derived sensitivity common for NV sensing. The projected sensitivity is based on

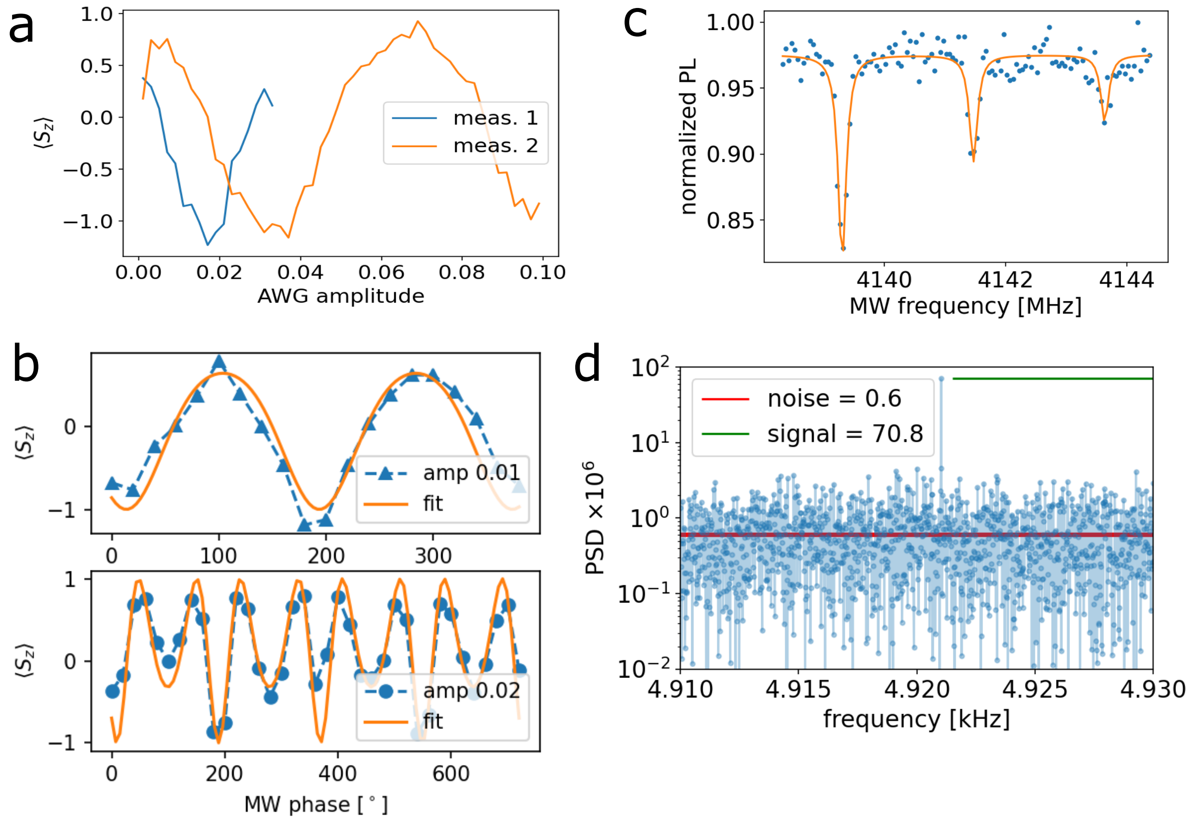

Supplementary Figure 1: Data for sensitivity estimation of pulsed Mollow scheme. **a** Sequence response as a function of signal amplitude. Two measurements with different starting phase give ambiguous results. **b** Initial phase sweep of the MW-signal with an AWG amplitude of 0.01 (top) and 0.02 (bottom) with dynamical decoupling (CMPG-20). The extracted sensor phase pickup is $\Phi_{\mathrm{sensor}} = 2.26 \pm 0.08$ radians for amplitude 0.01 and $\Phi_{\mathrm{sensor}} = 5.04 \pm 0.07$ radians for amplitude 0.02. **c** ODMR spectrum with ^{14}N hyperfine splitting and hyperpolarization. The contrast depending on the hyperfine state is $\kappa_{+1} = 0.161$, $\kappa_0 = 0.083$ and $\kappa_{-1} = 0.052$. **d** Example of a power spectral density of one measurement series with 10^6 measurements.

a shot noise limited readout with single quantum sensor, as for phase synchronous AC-sensing:

$$\bar{S} = \bar{n} \tag{31}$$

$$\delta S = \sqrt{\bar{n}} \tag{32}$$

$$S(B) = \bar{n}\left(1 + \frac{\kappa}{2}\sin\left(\frac{1}{2}\gamma B\tau\frac{2}{\pi}\right)\right) \tag{33}$$

$$\frac{\partial S}{\partial B} = \bar{n}\frac{1}{2}\kappa\frac{\gamma\tau}{\pi} \tag{34}$$

$$B_{min} = \delta S\frac{2\pi}{\bar{n}\kappa\gamma\tau} \tag{35}$$

$$\eta = B_{min}\cdot\sqrt{\tau} = 2\pi\frac{1}{\kappa\gamma\sqrt{\tau\bar{n}}} \tag{36}$$

$$\eta = \frac{2\pi}{0.083\cdot 2\pi\cdot 28\,\mathrm{GHz/T}\,\sqrt{68\,\mu\mathrm{s}\cdot 0.14}} \approx 141\,\mathrm{nT}/\sqrt{\mathrm{Hz}}, \tag{37}$$

with the average number of photons $\bar{n} = 0.14$, the signal S, the standard deviations of the signal δS, the contrast $\kappa = 0.083$ (central electron spin transition), the gyromagnetic ratio $\gamma = 28\mathrm{GHz/T}$, the magnetic field B, sensing time $\tau = 68\mu\mathrm{s}$.

The estimated sensitivity is by a factor of $\sqrt{2}$ smaller than the extracted sensitivity. This can be explained by considering the definition of the sensitivities from the extraction and the derivation. In the following we analyze the signal-to-noise ratio in the power spectral density. Firstly, we consider the noise. The noise is a white noise process with standard deviation σ. The auto-correlation of a white noise process is a delta function which Fourier transformed becomes a constant with σ^2. Therefore the noise floor in the power spectral density is σ^2. Secondly, we consider the signal which for our case is a non-decaying sine wave. The auto-correlation is given in the main text Equation 7, as $C(n) \approx \frac{1}{2}M\Phi^2\cos(\delta\omega Tn)$. When we perform the Fourier transform of this correlation function we get a signal height of $\frac{1}{2}M\Phi^2$. We define our sensitivity as the $SNR_{\mathrm{psd}}(\Phi_{\mathrm{min}}) = 1$, therefore our minimal sensor pickup phase is $\Phi_{\mathrm{min}} = \sqrt{2}\sigma/\sqrt{M}$. Common NV sensitivity estimations assume an optimal working point with slope 1 and measure at the same point for all measurements. Therefore the minimal detectable phase is given by

$\Phi_{\text{min}} = \sigma/\sqrt{M}$, a factor of $\sqrt{2}$ smaller compared to the extracted sensitivity for heterodyne sensing.

Supplementary Note 6: Linewidth over Correlation Time

In the main text we showed, for the dressed systems, the Fourier spectrum of the auto-correlation with very sharp peaks. In the following we analyzed the linewidth of these data sets by fitting a lorentzian to the peak and plotted the linewidth over the correlation length up to $N = 630957$. The label 3c refers to the data shown in the Mollow dressed system, where we studied three measurements. Dynamical decoupling (CPMG) with a RF sensing field, CPMG with a MW sensing field and the free interaction (FID) of the sensor with the MW sensing field. Together with the data set of Figure 5c in the main text, where we applied Floquet dynamics to sense MW fields. All data sets show a clear $1/N$ behavior. The y-axis is expressed in cycles per sample, which can be converted to frequency units of Hz by multiplying with the sampling rate $(1/T)$, with $T_{\text{RF}-\text{CPMG}} = 23.11\mu$s, $T_{\text{MW}-\text{FID}} = 4.5\mu$s, $T_{\text{MW}-\text{CPMG}} = 70.6815\mu$s and $T_{\text{MW}-\text{Floquet}} = 16.960\,\mu$s.

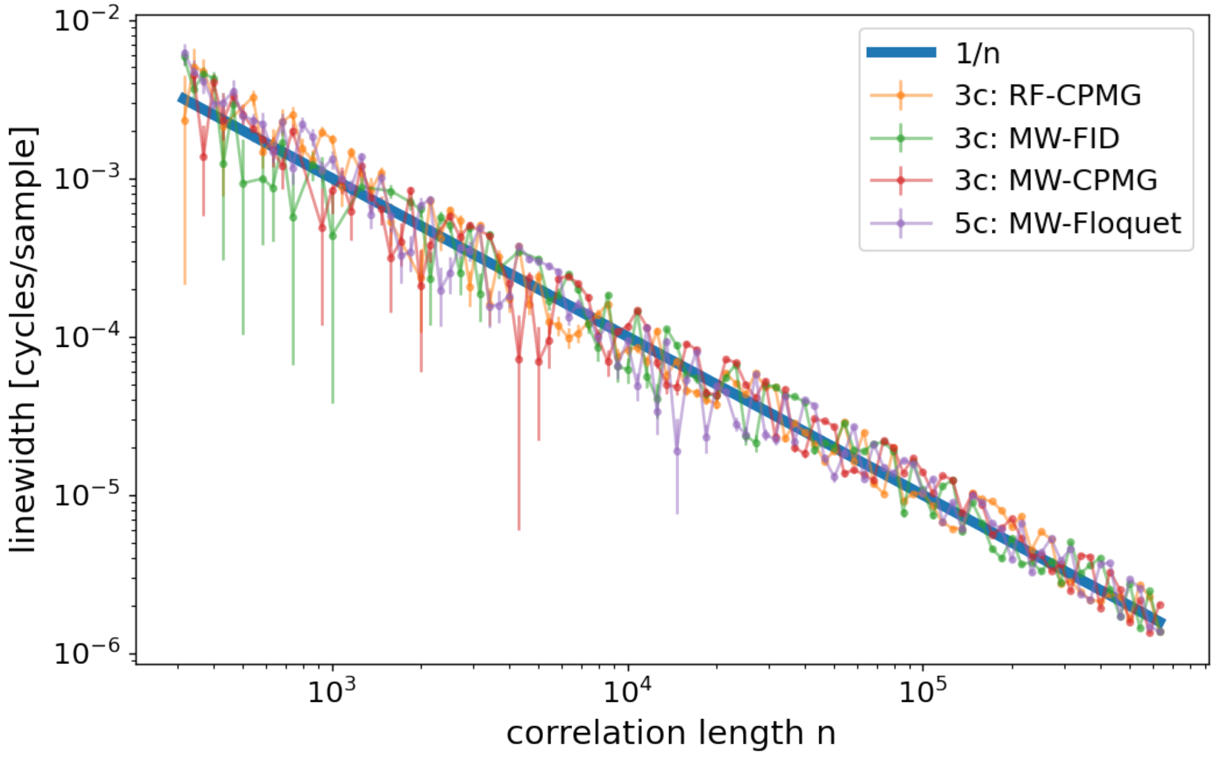

Supplementary Figure 2: Linewidth of the Fourier peak over the correlation length for the data sets. Data labeled with 3c shows the transitions of the Mollow dressed system with CPMG and without for RF and MW sensing fields. Data labeled with 5c shows the linewidth of the Fourier dressed system.

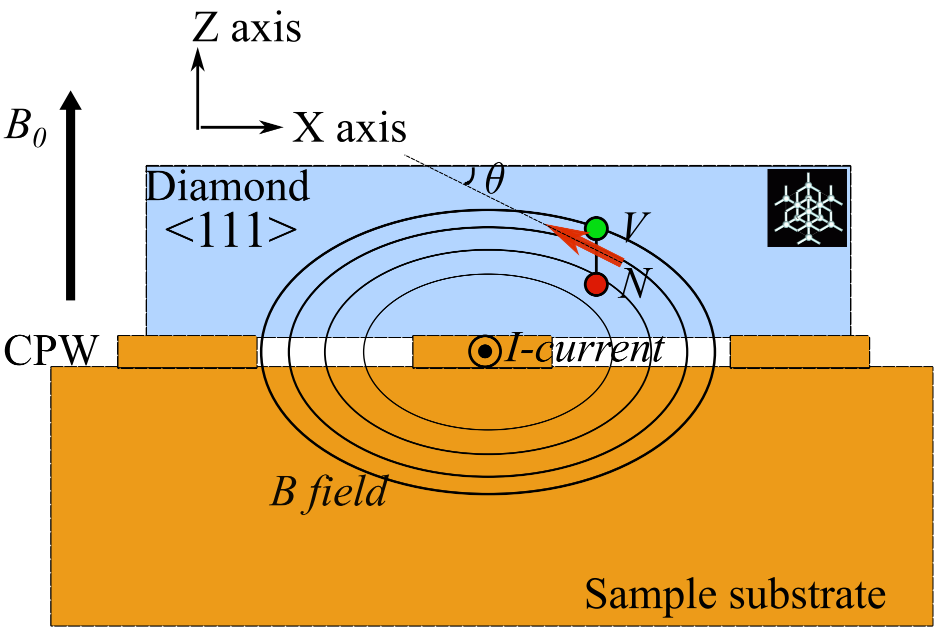

Supplementary Figure 3: Setup field and sample geometry. The NV center experiences a longitudinal and transverse MW/RF field component. The coplanarwave guide creates a longitudinal field (z) in the order of about $\leq 10\%$ of the transverse field (x).

Supplementary Note 7: Sample and MW Field Geometry

In Supplementary Note 4 we discuss the side wise component of the RF dressing fields. Here we further clarify the geometry of the sample and microwave structure. We estimate the ratio of transverse to longitudinal field to be $\leq 10\%$ from the Rabi frequency of ^{13}C as 20 kHz equal to $3\,\mathrm{mT}$ and the longitudinal field of approximate $0.1\,\mathrm{mT}$.

Supplementary Note 8: Variables

In Table 1 and Table 2 we list the variables used throughout the manuscript.

	**Probe Field**
ω	frequency of the probe field
$\Omega(t, \phi_0)$	General probe field
Ω_0	strength of the probe field
ϕ_0	initial phase of probe field
γB_{signal}	probe field as magnetic field
$\boldsymbol{\theta}$	unit vector of probe field direction
	**two-level System**
ω_s	TLS energy splitting
S_i	Spin operators
σ_i	Pauli matrices
U	evolution operator
$\langle S_z \rangle$	expectation Value
	1st **Rotating Frame**
$\Delta\omega = \omega_s - \omega$	Frequency difference between probe and TLS
Ω'	Effective Rabi frequency
	**Ext. Reference**
$\Omega_{\text{ref}}(t, \phi_{\text{ref}})$	Reference field general
$\|\psi_{\text{init}}(\phi_{\text{ref}})\rangle$	Initial state created by reference
	**Measurement and Synchronization**
Ξ_n	subscript n assigns to measurement number
$\delta\omega$	demodulation frequency
M	total number of measurements
t	global time
T	Time between measurements
S_n	Measurement outcome
$C(n)$	Autocorrelation
N	last point of auto correlation
τ	evolution time

Supplementary Table 1: Variables part 1

	**Dressing Fields**
Ω_{dressing}	Dressing fields switching the interaction
Ω_{dd}	Mollow: splitting
$\Omega_{dd} = \frac{\pi}{\tau_{dd}}$	Mollow: time between pulses
Ω_{rf}	Floquet: RF driving strength
m	Floquet: quantum number of Floquet states
Δm	Floquet: transitions between floquet states
x	Floquet: ratio of frequencies of RF
$P_{\Delta m}$	Rabi frequency reduction factor
J_i	Besselfunction first kind of i-order
	**Experimental Variables**
ω_1, ω_2	Two frequency signal
Ω_1, Ω_2	Two signal amplitudes
$\Delta\omega_1$, $\Delta\omega_2$	Two frequency difference relative to TLS
Ω_{Rabi}	Rabi frequency
	**NV specifics**
T_2^*	Decoherence FID
$T_{1,\rho}$	Lifetime in the rotating frame
T_1	Lifetime of the TLS
	**Sensitivity Estimation**
η	sensitivity
Φ_{Sensor}	sensor phase pick-up absorption
Φ_{exp}	sensor phase pick-up heterodyne experiment
Φ_{min}	minimal detectable sensor phase
B_{min}	minimal detectable field value
κ	Contrast

Supplementary Table 2: Variables part 2

Supplementary References

[1] Schmitt, S. *et al.* Submillihertz magnetic spectroscopy performed with a nanoscale quantum sensor. *Science* **356**, 832–837 (2017).

[2] Degen, C. L., Reinhard, F. & Cappellaro, P. Quantum sensing. *Reviews of modern physics* **89**, 035002 (2017).

[3] Ashhab, S., Johansson, J., Zagoskin, A. & Nori, F. Two-level systems driven by large-amplitude fields. *Physical Review A* **75**, 063414 (2007).

[4] Childress, L. & McIntyre, J. Multifrequency spin resonance in diamond. *Physical Review A* **82**, 033839 (2010).

[5] Ramsey, N. F. Resonance transitions induced by perturbations at two or more different frequencies. *Phys. Rev.* **100**, 1191–1194 (1955). URL `https://link.aps.org/doi/10.1103/PhysRev.100.1191`.

[6] Bloch, F. & Siegert, A. Magnetic resonance for nonrotating fields. *Phys. Rev.* **57**, 522–527 (1940). URL `https://link.aps.org/doi/10.1103/PhysRev.57.522`.
